# Long non-coding RNA AC026166.2-001 inhibits cell proliferation and migration in laryngeal squamous cell carcinoma by regulating the miR-24-3p/p27 axis

**DOI:** 10.1038/s41598-018-21659-5

**Published:** 2018-02-20

**Authors:** Zhisen Shen, Wenjuan Hao, Chongchang Zhou, Hongxia Deng, Dong Ye, Qun Li, Lexi Lin, Bing Cao, Junming Guo

**Affiliations:** 1 Department of Otorhinolaryngology and Head and Neck Surgery, the Affiliated Lihuili Hospital, Medical School of Ningbo University, Ningbo, 315040 China; 2 Department of Biochemistry and Molecular Biology, Zhejiang Key Laboratory of Pathophysiology, Medical School of Ningbo University, Ningbo, 315211 China

## Abstract

Long non-coding RNA (lncRNA) AC026166.2-001 was found to be down-regulated in laryngeal squamous cell carcinoma (LSCC) tissues and metastatic neck lymph nodes. Decreased AC026166.2-001 was associated with poorer prognosis and may act as a novel biomarker for LSCC patients. In this study, AC026166.2–001 was overexpressed by a lentivirus vector and down-regulated by a small interfering RNA (siRNA). The results of real-time cell analysis (RTCA) and a plate colony formation assay showed that AC026166.2–001 inhibited LSCC cell proliferation and the clone-forming capacity. Cell cycle distribution and related protein changes were measured by flow cytometry. AC026166.2–001 arrested the cell cycle at the G1 phase and induced apoptosis. In addition, AC026166.2–001 decreased cell migration as measured by wound healing assays and transwell migration assays. Moreover, luciferase reporter assay and Western blotting results suggested that AC026166.2–001 acts as a sponge of miR-24-3p and regulates the expression of p27 and cyclin D1. The *in vivo* results showed that AC026166.2–001 significantly suppressed the growth of LSCC xenografts and promoted apoptosis. We validated the molecular mechanisms underlying AC026166.2–001 in LSCC. This is the first report of AC026166.2–001 acting as a tumor suppressor in LSCC by regulating the miR-24-3p/p27 axis.

## Introduction

Laryngeal squamous cell carcinoma (LSCC) accounts for approximately 90% of all malignant tumors of the larynx and is the second most prevalent malignancy of the respiratory system. In the United States, the estimated incidence was 13,360 new cases and 3,660 estimated deaths per year in 2017; the incidence and mortality of males are approximately 3.8- and 4.1-times higher, respectively, than those of females^1^. In China, the incidence of LSCC was 26,400 and mortality reached 14,500 in 2015^2^. In terms of the epidemiological characteristics, smoking, drinking, occupational factors, air pollution, some male hormones, human papilloma virus (HPV), and gastroesophageal reflux disease are high risk factors related to the occurrence and development of laryngeal cancer^3–7^. The larynx plays a key role in breathing, swallowing and phonation, with the early symptoms of LSCC, such as hoarseness, dysphagia and cervical lymph node metastasis, being so common that they may be easily ignored. The treatment modalities for LSCC have changed significantly over the past 10 years, but significant challenges remain in improving patient’s survival rate and life quality after treatment^8^. Thus, safer and more noninvasive therapies are required.

In essence, tumors are genetic diseases with occurrence and development closely related to gene mutation, deletion and abnormality. As is well-known, only 2% of the human genome encodes genes, while the remainder consists of non-coding genes^9^. Non-coding RNAs have long been considered to be non-functional “trash”, but recent evidence indicates that lncRNAs could play a critical role in cellular function and disease processes, including transcription, mRNA stability, translation, alternative splicing, and protein-protein interactions^10^. This may be related to their ability to interact with DNA, RNA, or proteins to regulate gene expression^11^. Presently, there is significant evidence showing that lncRNAs can act as oncogenes or tumor suppressor genes.

lncRNA AC026166.2–001 is a reverse strand with a length of 344 nt. The *AC026166*.*2–001* (ENSG00000233026) gene is also called *mitochondrially encoded cytochrome c oxidase I pseudogene 5* (*MTCO1P5*), which is a known, processed pseudogene located on chromosome 3p25.2 and only has one transcript. In our previous work, we found that AC026166.2–001 was down-regulated in LSCC tissues and cervical lymph nodes^12^. Additionally, decreased expression of AC026166.2–001 in LSCC tissues indicated poor prognosis. Overall, AC026166.2–001 can serve as a novel biomarker and potential therapeutic target for LSCC, which also can be an independent prognostic factor for LSCC survival^12^. The biological functions and molecular mechanisms of AC026166.2–001 have not been reported. In this study, we investigated the biological functions of AC026166.2–001 in LSCC, especially to explore its role in proliferation and metastasis.

## Results

### lncRNA AC026166.2–001 suppresses cell proliferation and colony formation in LSCC cells

**Figure 1:**
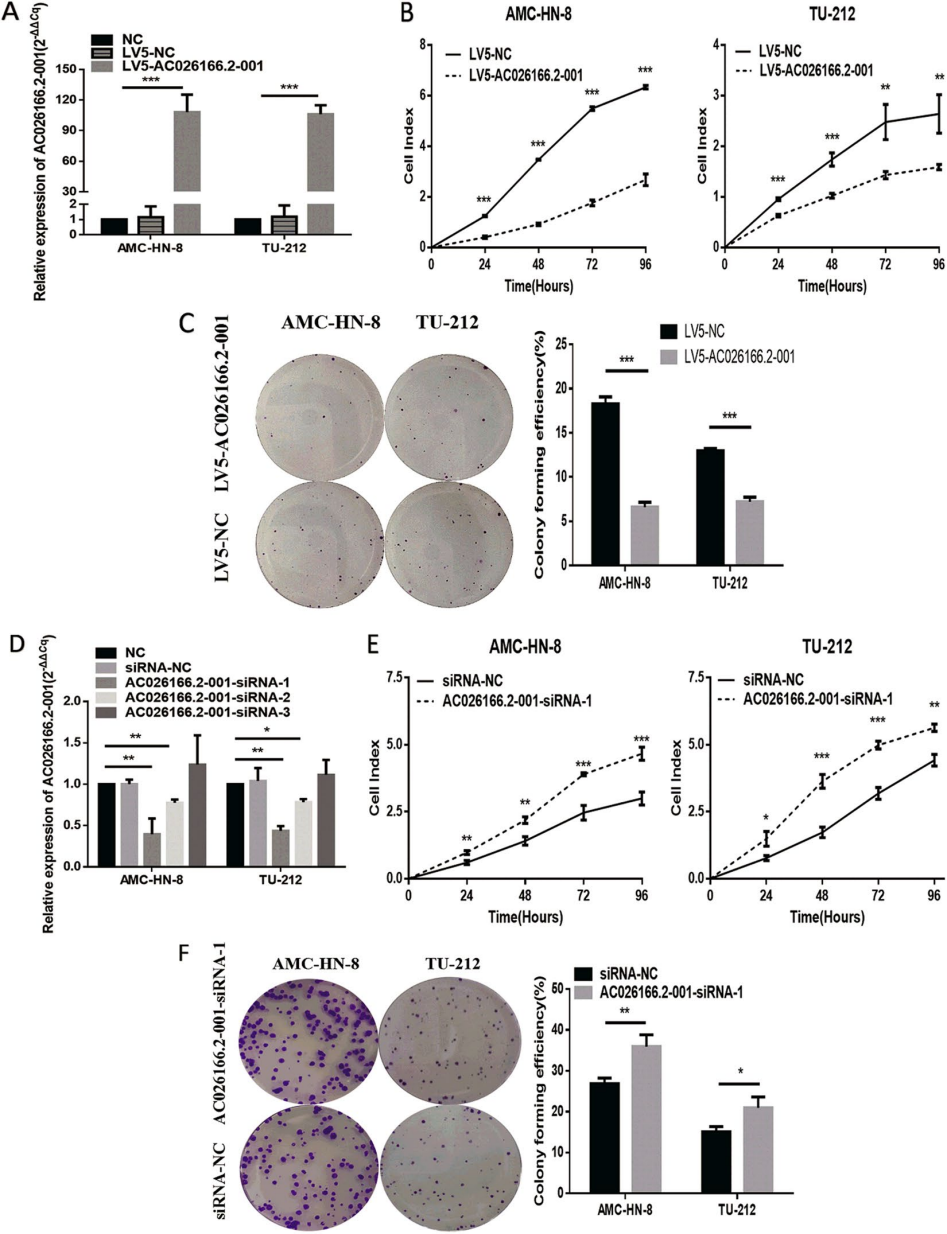
AC026166.2-001 inhibits cell proliferation and colony formation by LSCC cells. (**A**) Overexpression of AC026166.2-001 in LSCC cell lines was analyzed by qRT-PCR. (**B**) AC026166.2-001 significantly inhibited proliferation rates during a period of 96 h determined by RTCA. (**C**) AC026166.2-001 reduced the colony formation capacity of AMC-HN-8 and TU-212 cells. (**D**) AC026166.2-001 expression was examined in NC (non-transfected control) and siRNA transfected LSCC cells by qRT-PCR. (**E**) Cell proliferation was determined in siRNA-NC and AC026166.2-001-siRNA-1-transfected AMC-HN-8 and TU-212 cells by RTCA during a period of 96 h. (**F**) Colony formation capacity of siRNA-NC and AC026166.2-001-siRNA-1-transfected AMC-HN-8 and TU-212 cells.

Since the expression of AC026166.2–001 is down-regulated in LSCC tissues and metastatic neck lymph nodes^12^, to elucidate the role of AC026166.2–001 in tumor progression, we up-regulated AC026166.2–001 expression using the lentivirus vector (LV5-AC026166.2–001) or a negative control (LV5-NC) in AMC-HN-8 and TU-212 cells. After selection by puromycin, AC026166.2–001 in the two cell lines was up-regulated to levels reaching 108- and 106-times (LV5-AC026166.2–001 vs. LV5-NC; Fig. 1A). Then, we conducted real-time cell analysis (RTCA) and colony formation assays to identify the influence of AC026166.2–001 on LSCC cell proliferation. RTCA showed that the cell index (CI) of the AC026166.2–001 overexpressing cell lines decreased significantly (Fig. 1B). Furthermore, colony numbers of AC026166.2–001 overexpressed cells were significantly less than those transfected with LV5-NC (Fig. 1C). Both methods confirmed that when lncRNA AC026166.2–001 was overexpressed, cell proliferation was suppressed. To further elucidate the relationship between AC026166.2–001 and LSCC cell proliferation, we down-regulated AC026166.2–001 by transfection with three small interfering RNAs (siRNAs). Following a 24 h transfection, the expression of AC026166.2–001 was significantly decreased by AC026166.2–001-siRNA-1 and AC026166.2–001-siRNA-2, whereas the AC026166.2–001-siRNA-3 group was not significantly different compared with the siRNA-NC (Fig. 1D). Given the optimal transfection efficiency of AC026166.2–001-siRNA-1, we decided to select it for further experiments. The results showed that LSCC cells transfected with AC026166.2–001-siRNA-1 had a significant increase in the value of their CI compared with siRNA-NC transfection (Fig. 1E). We also found that the colony-forming ability of LSCC cells increased after treatment with AC026166.2–001-siRNA-1 (Fig. 1F).

### Influence of AC026166.2–001 on cell cycle and apoptosis

**Figure 2:**
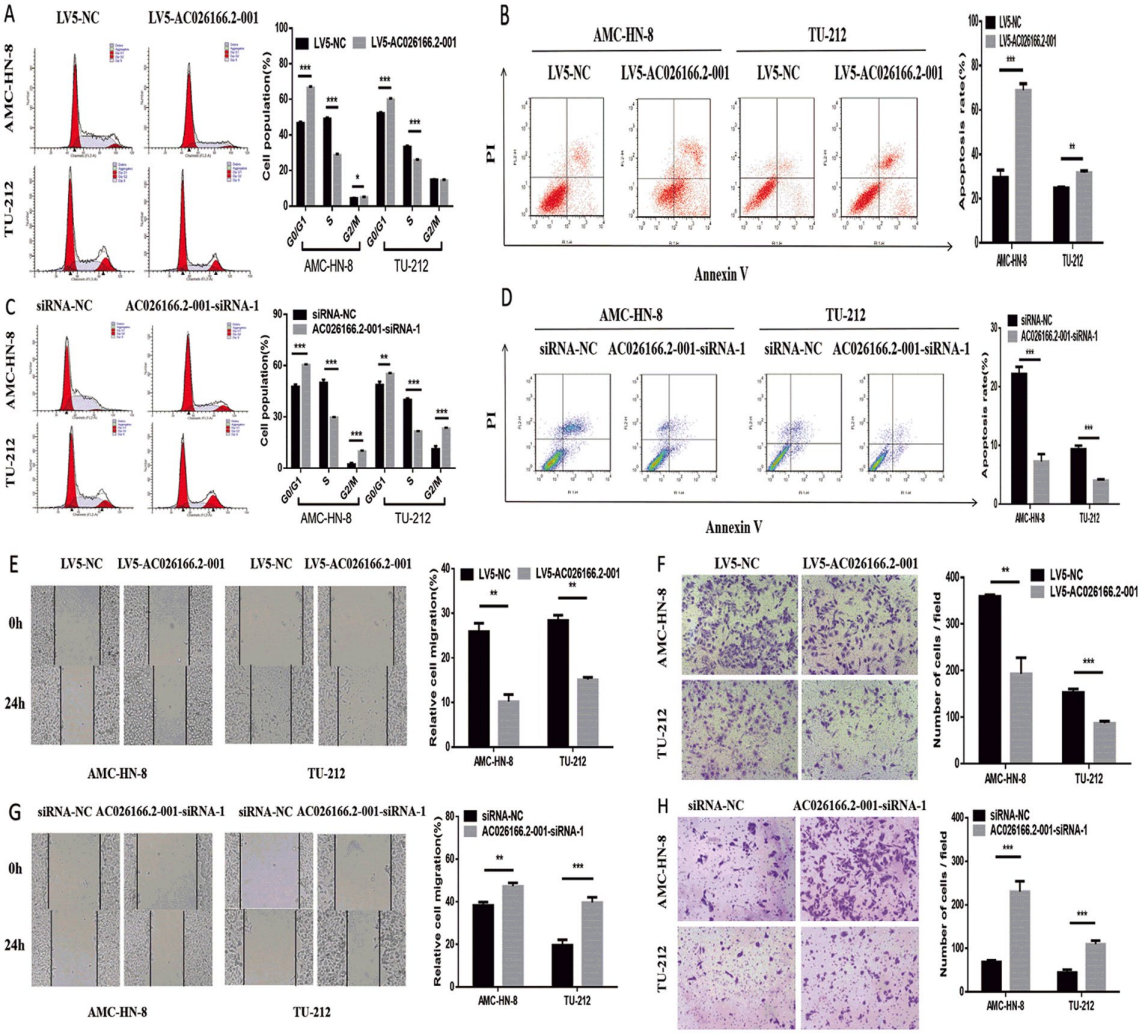
AC026166.2-001 suppresses cell migration and induces cell cycle arrest in the G0/G1 phase of LSCC cells. (**A**) Cell cycles determined in LV-5-NC and LV5-AC026166.2-001-transfected AMC-HN-8 and TU-212 cells were analyzed by flow cytometry. (**B**) Cell apoptosis determined in LV-5-NC and LV5-AC026166.2-001-transfected AMC-HN-8 and TU-212 cells was analyzed by flow cytometry using Annexin V/PI staining. (**C**) Cell cycles determined in siRNA-NC and AC026166.2-001-siRNA-1-transfected AMC-HN-8 and TU-212 cells were analyzed by flow cytometry. (**D**) After deletion of AC026166.2-001, cell apoptosis was measured by flow cytometry using Annexin V/PI staining. (**E**) Wound healing analysis and transwell migration assays (**F**) were used to determine cell migration in LV-5-NC and LV5-AC026166.2-001-transfected AMC-HN-8 and TU-212 cells (Magnification × 40). (**G**) Wound healing analysis and transwell migration assays (**F**) were used to determine cell migration in siRNA-NC and AC026166.2-001-siRNA-1-transfected AMC-HN-8 and TU-212 cells (Magnification × 40). Data are presented as the mean ± SD, *n* = 3. *P < 0.05, **P < 0.01, ***P < 0.001.

To further explore the potential regulatory mechanism of AC026166.2–001 on cell proliferation, cell cycle and apoptosis were measured by flow cytometry. The results showed that AC026166.2–001 arrested the cell cycle at the G0/G1 phase and decreased the cell cycle at the S phase (Fig. 2A). Moreover, the results of the apoptosis assay showed that the percentage of apoptotic cells in both AMC-HN-8 and TU-212 cells significantly increased (Fig. 2B). These data suggested that the up-regulation of AC026166.2–001 inhibited LSCC cell transition from the G0/G1 to S phase and promoted cell apoptosis. At the same time, compared with siRNA-NC transfected cells, AC026166.2–001 knockdown arrested the cell cycle at the S phase in both LSCC cell lines (Fig. 2C) and inhibited cell apoptosis (Fig. 2D). These results indicated that AC026166.2–001 inhibited the G1-S cell cycle progression and promoted cell apoptosis in LSCC.

### Role of AC026166.2–001 in LSCC cell migration

To further confirm the effect of lncRNA AC026166.2–001 on LSCC cell migration, we investigated cell migration in two ways. The first method involved wound healing analysis. We found that up-regulation of AC026166.2–001 significantly reduced the migration ability of LSCC cells (Fig. 2E). After down-regulating the expression of AC026166.2–001 by siRNA, the migration ability of cells was enhanced (Fig. 2F). With the second method, the results of transwell migration assays reached the same conclusion. The expression level of lnc-AC026166.2–001 was negatively correlated with the migration ability of LSCC cells (Fig. 2G,H).

### AC026166.2-001 may acts as a molecular sponge for miR-24-3p

**Figure 3:**
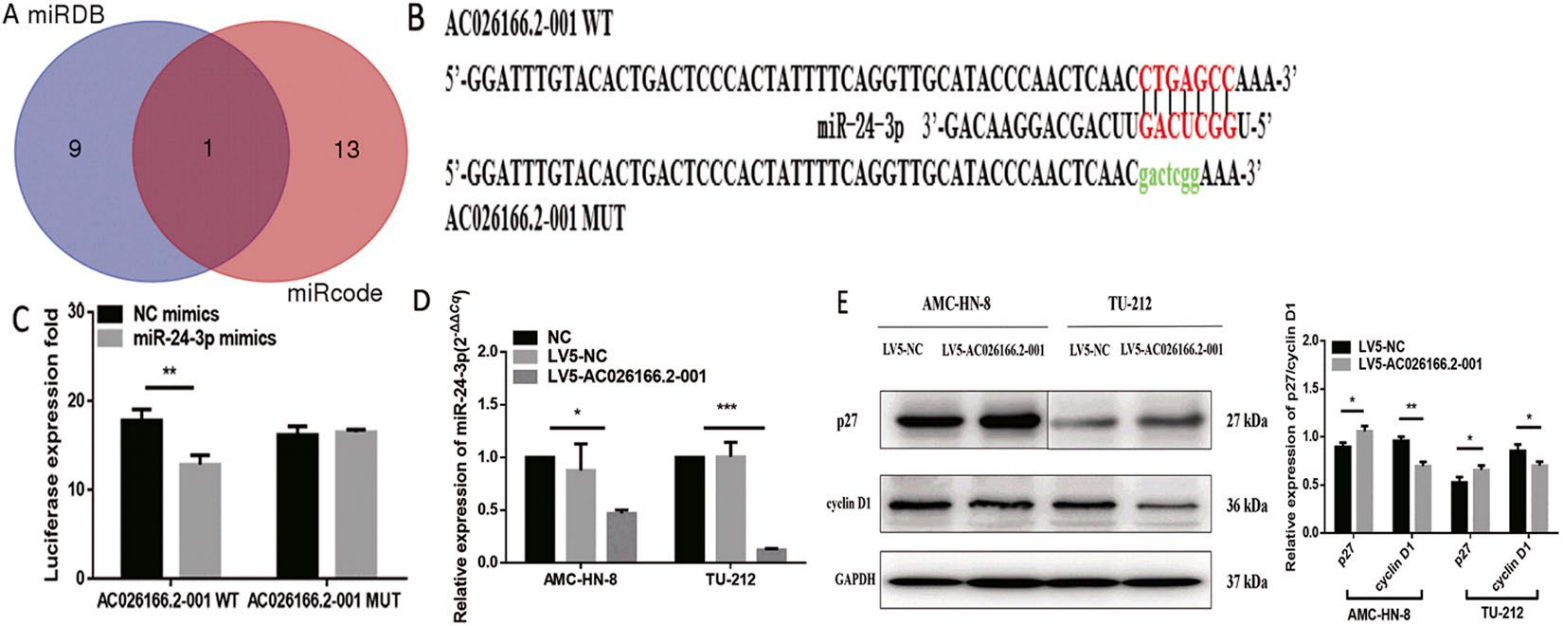
AC026166.2-001 targets miR-24-3p and promotes p27/Kip1 expression. (**A**) miRDB: miR-3127-5p, miR-1324, miR-3677-3P, miR-4261, miR-4496, miR-4330, miR-24-3p, miR-219a-2-3p, miR-4727-3p, miR-494-3p. miRcode: miR-190, miR-190a, miR-190b, miR-205, miR-205a, miR-205b, miR-23a, miR-23b, miR-23c, miR-23b-3p, miR-24, miR-24a, miR-24a, miR-24b, miR-24-3p. Both: miR-24-3p. (**B**) Bioinformatics analysis showed 7 bases in the matched binding sites between AC026166.2-001 and miR-24-3p. As shown above, binding sites or mutated sequence were used for creating firefly luciferase reporter constructs. (**C**) There are 4 groups (AC026166.2-001 WT/MUT 3′-UTR reporter + miR-24-3p mimics/miR-NC) for the luciferase reporter assay and results demonstrated that miR-24-3p inhibited the AC026166.2-001 WT but not the MUT. (**D**) After overexpression of AC026166.2-001, miR-24-3p was down-regulated in AMC-HN-8 and TU-212 cell lines. (**E**) Protein levels of p27, cyclin D1 in AMC-LHN-8 and TU-212 cells transfected with lentiviruses LV5-AC026166.2-001 or LV5-NC determined by Western blot analysis. For details, see Supplementary Figure S1.

It is increasingly evident that lncRNAs are involved in both normal physiology and tumorigenesis by competing with endogenous RNA (ceRNA). However, the functional roles and regulatory mechanisms of AC026166.2-001 as a ceRNA in LSCC remain unclear. We predicted its target sites and found that only miR-24-3p has the relevant binding sites (Fig. 3A). On the basis of these binding sites, we used AC026166.2-001 WT/MUT GP-miRGLO vectors cotransfected with miR-24-3p mimics or miR-NC (miRNA negative control) in 293 T to examine whether AC026166.2-001 combines with miR-24-3p (Fig. 3B). The results showed that overexpression of miR-24-3p significantly reduced the luciferase activity of AC026166.2-001 WT GP-miRGLO vectors but not of AC026166.2-001 MUT (Fig. 3C). Next, we hypothesized that AC026166.2-001 may act as a sponge for miR-24-3p. Quantitative RT-PCR was used to test our assumption and we found that miR-24-3p was down-regulated by AC026166.2-001 (Fig. 3D).

### Molecular mechanisms of AC026166.2-001 in cell proliferation

We evaluated the molecular mechanisms by which AC026166.2-001 inhibited cell transition from the G0/G1 to the S phase and the target of miR-24-3p. Western blot assays were used to measure the associated protein expression levels. Compared with LV5-NC-transfected cells, the expression of p27 was significantly elevated in LV5-AC026166.2-001 cells, while cyclin D1 expression decreased (Fig. 3E).

### Overexpression of AC026166.2-001 inhibits the growth of LSCC xenografts

**Figure 4:**
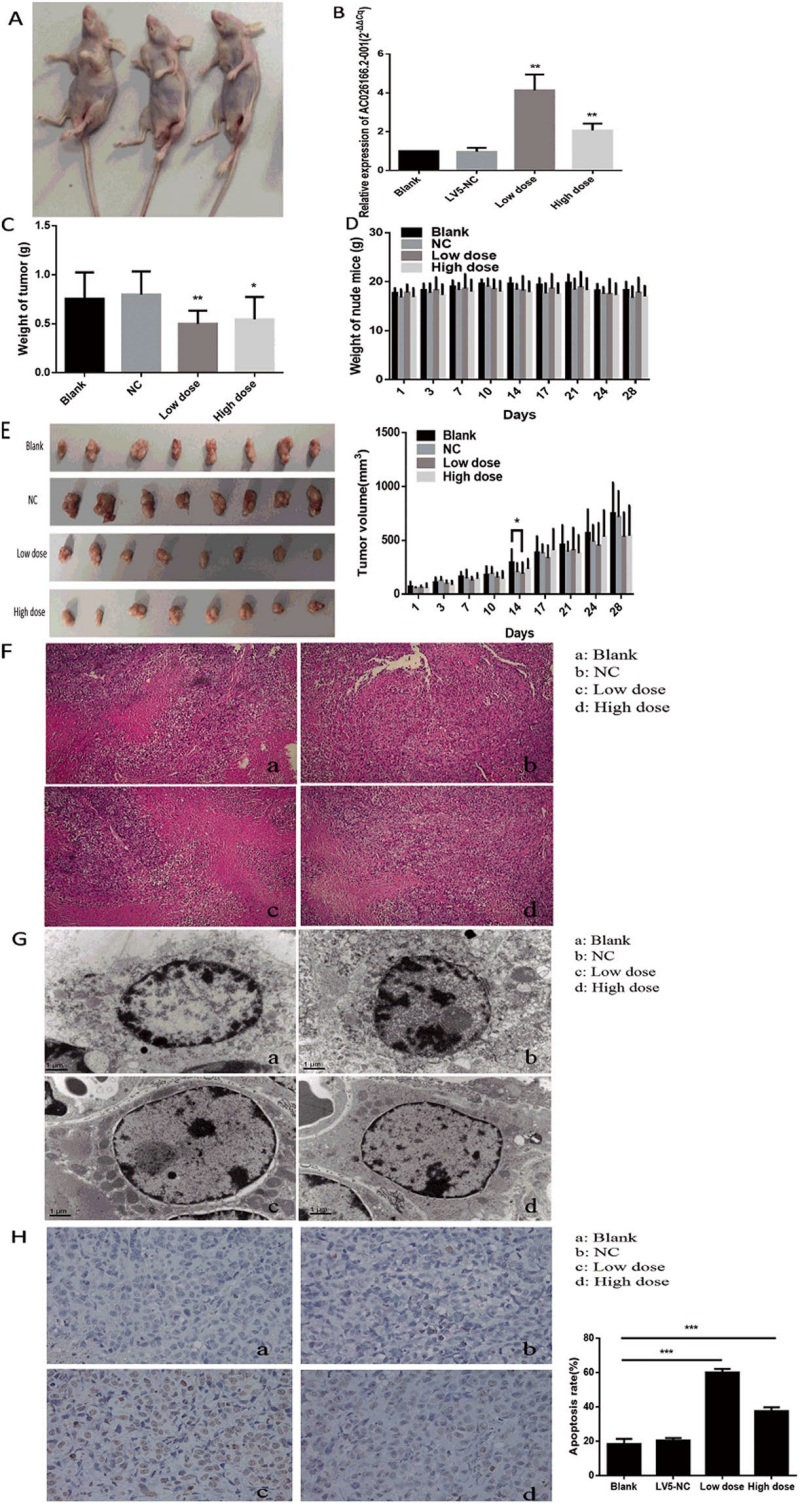
AC026166.2-001 inhibited the proliferation and induced the apoptosis of LSCC cells *in vivo*. (**A**) Xenograft mouse model. (**B**) Expression of AC026166.2-001 in 4 groups of xenograft tumor tissues analyzed by qRT-PCR. (**C**) Tumor weight in low-dose and high-dose AC026166.2-001-treated groups were significantly lower than the blank control group. (**D**) Weight of nude mice showed no significant difference during the experiment. (**E**) The tumor volume of the nude mice low-dose group was significantly smaller than the blank control group on the fourteenth day. (**F**) Pathological examination showed moderate differentiated squamous cell carcinoma and there was no significant difference between the groups (Magnification × 100). (**G**) a and b: Cells in AMC-HN-8 xenografts had normal morphology, c and d: Apoptotic morphologies were found in low-dose and high-dose AC026166.2-001-treated AMC-HN-8 xenografts (Magnification × 15000). (H) Paraffin-embedded tissue sections were analyzed by TUNEL assay. In LV5-AC026166.2-001-injected groups, the number of TUNEL + cells increased markedly compared with the blank and LV5-NC treatment (Magnification × 400).

In the study of LSCC, the above results indicated that AC026166.2-001 acts as a tumor suppressor *in vitro*. Furthermore, to provide evidence for the anti-oncogenic role of AC026166.2-001 *in vivo*, we used a xenograft mouse model (Fig. 4A). Eight BALB/c nude mice were tested in each group. A high or low dose of LV5-AC026166.2-001 or LV5-NC with transfection reagent was injected into the tumor. Final results showed that overexpression of AC026166.2-001 significantly inhibited tumor growth. The expression of AC026166.2-001 was up-regulated in the high- and low-dose LV5-AC026166.2-001-treated xenograft tumor tissues (Fig. 4B). The tumor weight of LSCC xenografts in both high- and low-dose LV5-AC026166.2-001-treated groups was significantly lower than in the control group. The rate of the inhibitory effect ranged from 29.7% to 35.6% (Fig. 4C). During the experimental process, there was no significant difference in the weight of nude mice in these groups (Fig. 4D). In addition, on the fourteenth day, the tumor volume of nude mice treated with the low-dose LV5-AC026166.2-001 was significantly smaller than that in the control group (Fig. 4E). All pathological findings showed that the tumor consisted of moderately differentiated squamous cell carcinomas with necrosis and inflammatory cell infiltration in some areas. This proved the success of the xenograft mouse model (Fig. 4F).

### AC026166.2-001 induces apoptosis of LSCC cells *in vivo*

Apoptotic cells have special structural features as determined by transmission electron microscopy. Typical apoptotic morphology of tumor cells includes homogeneous condensation of chromatin clustered to one side at the periphery of the nuclei, appearing as crescents or circular bodies. While the cytoplasm is concentrated, the endoplasmic reticulum is loose and fused with the membrane. In the late stage of apoptosis, the nuclei are cleaved into fragments and produce apoptotic bodies showing an incomplete morphology of the membrane and organelles. Our results showed that typical apoptotic morphology was present in the LV5-AC026166.2-001-treated group but not in the control group (Fig. 4G). The TUNEL assay (terminal deoxynucleotidyl transferase-mediated dUTP-biotin nick end labeling assay) is a common method used to detect apoptotic cells. In this assay, when the genomic DNA is cleaved, the exposed 3′-OH can be biotin-labeled with dUTP (Biotin-dUTP) catalyzed by terminal deoxynucleotidyl transferase (TdT). With the biotin label, DNA cleavage sites were detected by reaction with streptavidin-HRP. When visualized by DAB, apoptotic cells could be detected under light microscopy as a brown color. The results of the TUNEL assay showed that the number of apoptotic-positive cells in both LV5-AC026166.2-001-treated groups was significantly higher than that in the LV5-NC group and blank control group (Fig. 4H). Thus, AC026166.2-001 induction of LSCC cell apoptosis was also demonstrated *in vivo*.

## Discussion

lncRNAs, as its name implies, are long RNA transcripts that do not encode proteins. The length of lncRNAs is commonly defined as longer than 200 nucleotides, which primarily serves as a cutoff value to distinguish the known short ncRNA (such as miRNAs, siRNA, Piwi-interacting RNA and others). Expression of lncRNAs has a certain specificity, which is related to the type of cells, tissues, diseases and stages of the disease^13–15^. The mechanisms of lncRNA depend on interactions with cellular macromolecules. For example, combinations of lncRNA with chromatin can regulate local chromatin architecture or recruit regulatory molecules to specific loci, interactions of lncRNA with proteins can promote or suppress the formation of protein complexes, and lncRNA interactions with mRNA can recruit protein to affect splicing, mRNA stability, and translation or can adsorb miRNA to liberate target mRNA^16^.

With an increasing focus on the roles of lncRNAs as oncogenes and tumor suppressors in cancers, recent studies have reported that a large number of lncRNAs can serve as biomarkers to evaluate cancer status and provide prognostic values or can serve as molecular targets in cancer therapy, including lung cancer, gastric cancer, colorectal cancer and others^17–26^. In LSCC, a large number of lncRNAs have been reported to play important roles in tumorigenesis. Li *et al*. showed that homeobox (HOX) transcript antisense RNA (HOTAIR) overexpressed in LSCC acts as an oncogene for promoting *PTEN* methylation^27^. In addition, a serum exosomal miR-21 and HOTAIR combined-examination may be useful as a serum biomarker in LSCC^28^. lncRNA H19 promoted LSCC progression via miR-148a-3p and the DNA methyltransferase enzyme (DNMT1)^29^. In addition, NEAT1 plays an oncogenic role in the tumorigenesis of LSCC and may serve as a potential target for therapeutic interventions^30^.

In our previous study, we established the first complete lncRNA expression microarray profile in seven pairs of LSCC tissues, which can be obtained from the National Center for Biotechnology Information (NCBI) Gene Expression Omnibus (GEO) by searching the accession number GSE59652 (http://www.ncbi.nlm.nih.gov/geo/query/acc.cgi?acc = GSE59652)^12^. To verify the microarray result that AC026166.2-001 is down-regulated in LSCC tissues, we expanded the sample size to 87 paired surgical samples and metastatic neck lymph nodes. The final results showed that AC026166.2-001 is down-regulated in laryngeal cancer tissues compared with adjacent tissues and is reduced in metastatic cervical lymph nodes. There was no distinct relationship between AC026166.2-001 and clinicopathological features; however, survival data suggested that lower levels of AC026166.2-001 in tumor tissues were significantly correlated with poor prognosis. Additionally, AC026166.2-001 could be a potential biomarker for LSCC diagnosis as the area under the ROC curve was 0.65. Although down-regulated AC026166.2-001 has been demonstrated to contribute to the development of LSCC, its functional role in LSCC remains largely unknown.

In this study, we demonstrated that AC026166.2-001 can serve as a tumor suppressor in LSCC progression both *in vitro* and *in vivo*. First, we used the LV5-AC026166.2-001 lentivirus to up-regulate lncRNA in LSCC cells. To explore whether AC026166.2-001 plays a role in proliferation in LSCC, we performed RTCA and colony formation analysis *in vitro*. Our data showed that AC026166.2-001 suppressed cell proliferation (Fig. 1B and C). In addition, we determined the function of AC026166.2-001 in LSCC cells by testing loss of function. Cell proliferation and colony formation capacity were promoted in AC026166.2-001-siRNA-1-treated AMC-HN-8 and TU-212 cells (Fig. 1E and F). Cell cycle and apoptosis were assessed to further investigate the biological functions of AC026166.2-001 in LSCC cells. The results indicate that up-regulation of AC026166.2-001 induced cell cycle arrest in the G0/G1 phase and promoted apoptosis in LSCC cells (Fig. 2A and B). In contrast, AC026166.2-001 deletion showed the opposite result, with cell cycle reduced at the G0/G1 phase and increased at the S and G2/M phases (Fig. 2C). The cell apoptosis rate (%) decreased in AC026166.2-001-siRNA-1-treated AMC-HN-8 and TU-212 cells (Fig. 2D). Furthermore, experiments in BALB/c mice xenografts reached the same conclusion showing that AC026166.2-001 suppressed tumor growth *in vivo* and induced tumor cell apoptosis (Fig. 4C,G,H). After AC026166.2-001 overexpression, the xenograft tumor weight was significantly reduced (Fig. 4C), but the tumor volume (Fig. 4E) was not significantly different from the control group. We speculate that this may have been due to the presence of many necrotic and apoptotic cells in the tumor because a large number of necrotic and apoptotic cells were found in the tumor by TUNEL assay (Fig. 4H). Because AC026166.2-001 was significantly down-regulated in neck lymph nodes, we questioned if AC026166.2-001 might be involved in the metastasis of laryngeal cancer. The results of the wound-healing analysis and transwell migration assays confirmed this hypothesis. Cell migration capacity was inhibited in LV5-AC026166.2-001-treated AMC-HN-8 and TU-212 cells (Fig. 2E and F) but was promoted in AC026166.2-001-siRNA-1-treated cells (Fig. 2G and H). Taken together, our data strongly suggest that AC026166.2-001 has an inhibitory effect on LSCC cells.

The underlying molecular mechanisms of lncRNAs in cancer are complicated. The mechanisms include chromatin silencing, transcriptional regulation, post-transcriptional regulation or competitive binding sites of miRNAs to reduce translation or degradation of mRNA. Among them, the function of a competitive endogenous RNA (ceRNA) is gaining notable attention. Both mirDB and mircode programs predicted that miR-24-3p may be a target of AC026166.2-001 (Fig. 3A). miRNAs can typically bind the 3′-UTR (untranslated region) of the mRNA and provide a biological function in the form of a miRISC (miRNA-induced silencing complex)^31^. Studies have reported that miR-24-3p functions as an oncogene in multiple cancers including bladder cancer, hepatocellular carcinoma, head and neck squamous cell carcinoma^32–34^. In this study, a luciferase reporter assay revealed that miR-24-3p is a direct target gene of AC026166.2-001 (Fig. 3C). We also showed that LV5-AC026166.2-001 decreased miR-24-3p levels (Fig. 3D). Because AC026166.2-001 induced cell cycle arrest in the G1 phase and AC026166.2-001 served as a ceRNA of miR-24-3p (Figs 1C and 3C), we suspected that targets of miR-24-3p may be related to cell cycle progression. Target analysis predicted that p27 may be a target of miR-24-3p and that has been validated in human breast cancer and Hodgkin’s lymphoma^35,36^. As a critical regulator of the G1/S phase transition, p27 is pivotal in cell cycle progression. To the best of our knowledge, p27 is one of the cyclin-cyclin-dependent kinase inhibitors (CDIs). It exerts its functions by binding with cyclin-cyclin-dependent kinase (CDK) complexes to regulate the transition from the G1 to the S phase. Reduced expression of p27 has been reported to be associated with both reduction of disease-free patients and overall survival in LSCC^37^. It has been reported that the cyclin D1^+^/p27^−^ phenotype indicates poor prognosis for LSCC patients^38^. The results of Western blots revealed that overexpression of AC026166.2-001 up-regulates p27 and down-regulates cyclin D1 (Fig. 3E), which may be two of the molecular mechanisms of AC026166.2-001 in LSCC. Our study revealed that lncRNA AC026166.2-001 acted as a decoy of miR-24-3p to regulate p27 expression (Fig. 3C and E). However, further studies are needed to determine the role of miR-24-3p in LSCC.

In summary, this is the first study demonstrating that AC026166.2-001 acts as a tumor suppressor in LSCC. AC026166.2-001 inhibited proliferation and promoted apoptosis of LSCC cells *in vitro* and *in vivo*. The molecular mechanism of AC026166.2-001 may be associated with the miR-24-3p/p27 pathway. AC026166.2-001 may serve as a potential therapeutic target for the treatment of LSCC.

## Methods

### Cell culture and transfection

The LSCC cell line AMC-HN-8 was obtained from the BeNa Culture Collection (Jiangsu, China) and TU-212 was obtained from Jennio Biological Technology (Guangzhou, China). AMC-HN-8 cells were maintained in Dulbecco’s modified Eagle’s medium (DMEM) with high glucose (Hyclone, Logan, Utah, USA) and 10% fetal bovine serum (FBS) (PAN-Biotech, Adenbach, Germany). TU-212 cells were cultured inRPMI-1640 (Hyclone) with 10% FBS. Cells were cultured in a humidified 5% CO_2_ atmosphere in a 37 °C incubator (Heal Force 90, Hong Kong, China). Cells were counted using a TC10 Automated Cell Counter (Bio-Rad, Hercules, CA, USA). Overexpressed lentiviruses were purchased from GenePharma (Shanghai, China). Lentiviruses with polybrene (5 µg/ml) were used for LSCC cell line transfection. Stably transfected cells were selected by puromycin. Overexpressed AC026166.2-001 lentiviruses were named LV5-AC026166.2-001 and the empty lentiviral vector LV5-NC was used as a control. Three small interfering RNAs (siRNAs) were synthesized by Genema (Shanghai, China) for the AC026166.2-001 knockdown assay in AMC-HN-8 and TU-212 cells: AC026166.2-001-siRNA-1 (sense sequence: 5′-GGUUGCAUACCCAACUCAATT-3′, antisense sequence: 5′-UUGAGUUGGGUAUGCAACCTT-3′), AC026166.2-001-siRNA-2 (sense sequence: 5′-CCUACUGCCCUGAUGCAUATT-3′, antisense sequence: 5′-UAUGCAUCAGGGCAGUAGGT-3′), and AC026166.2-001-siRNA-3 (sense sequence: 5′-GCCCAUCACCAUAUCAUACTT-3′, antisense sequence: 5′-GUAUGAUAUGGUGAUGGGCTT-3′). The siRNA-NC (sense sequence: 5′-UUCUCCGAACGUGUCACGUTT-3′, antisense sequence: 5′-ACGUGACACGUUCGGAGAATT-3′) was used as a nonspecific scrambled control. The siRNA transfection was conducted with Lipofectamine 2000 (Thermo Fisher Scientific, MA, USA) in 6-well plates with 10^5^ cells for 24 h transfection.

### RNA extraction

Total RNA was extracted from cells and xenograft tumor tissues by the TRIzol reagent (Invitrogen, Carlsbad, CA, USA) in accordance with the manufacturer’s instructions. In the process of extraction, all the steps were carried out in an RNase-free condition. Total RNA was dissolved in RNase-free water, treated with DNase, and preserved at −80 °C until use. The concentration of total RNA was measured with a DS-11 Plus Spectrophotometer (DeNovix, Wilmington, Delaware, USA) and RNA purity was assessed according to the ratio of A260/A280 and A260/A230.

### Reverse transcription reaction and quantitative real-time PCR

Quantitative real-time reverse transcription-polymerase chain reaction (qRT-PCR) was carried out to measure the expression of lncRNA in LSCC cell lines and xenograft tumor tissues. The GoScript^TM^ Reverse Transcription System kit (Promega, Madison, WI, USA) was used to reverse-transcribe total RNA into cDNA. Next, cDNA was used as a template with the GoTaq^®^ qPCR Master Mix kit (Promega, Madison, WI, USA) and the reaction was monitored in an Mx3005P QPCR System (Stratagene, La Jolla, CA, USA) to detect expression levels of lncRNA. In this study, the housekeeping gene glyceraldehyde-3-phosphate dehydrogenase (GAPDH) was used as a reference to normalize the threshold cycle (*C*q) value. Primers were synthesized by Sangon Biotech (Shanghai, China) and their sequences were as follows: AC026166.2-001: sense: 5′-CCACGATGTTTCCTTGGTTTA-3′; antisense: 5′-ATTTGTGATTGAAAGGTTCTACTAC-3′, and GAPDH: sense: 5′-ACCCACTCCTCCACCTTTGAC-3′; antisense: 5′-TGTTGCTGTAGCCAAATTCGTT-3′.

The detection of miR-24-3p in LSCC cell lines was carried out with the miRcute Plus miRNA first-strand cDNA synthesis kit and the miRcute miRNA qPCR detection kit (SYBR Green) (TianGen, Beijing, China). U6 was used as an RT-PCR reference. The reverse primer was contained in the miRcute miRNA qPCR Detection Kit and forward primer sequences were as follows: miR-24-3p: 5′-TGCGGTGGCTCAGTTCAGCAGGAAC-3′; U6: 5′-TGCGGGTGCTCGCTTCGGCAGC-3′.

All experiments were performed in triplicate. Relative quantification of gene expression was calculated by the 2^−ΔΔ*C*q^ method.

### Cell proliferation and colony formation analysis

Real-time cell analysis (RTCA) (ACEA Biosciences, San Diego, CA, USA) monitors cell growth in real-time. Cell Index (CI) is regarded as an indicator of cell proliferation. LSCC cells were cultured in the E-plate 96 (ACEA Biosciences, San Diego, CA, USA) and the CI was measured for 96 h in total.

For colony formation, cells were trypsinized into single cells and seeded in the 6-well plates at a density of 500 cells/well. After 10 days of culture, cell clones that had formed from individual cells were directly observed by eye then fixed with 4% paraformaldehyde for 15 min and stained with 0.1% crystal violet (Sigma, Germany) solution for 30 min. The colony-forming efficiency (%) was calculated by the ratio of colony number and cell seeding number.

### Cell cycle analysis

First, cells were serum-starved to synchronize the cell cycle. Cells were then collected, washed in phosphate-buffered saline (PBS) and fixed in 70% ethanol at −20 °C for 24 h. After fixing, cells were rehydrated with pre-cooled PBS, stained with a DNA staining solution (Cell Cycle Staining Kit, Multi Sciences, Hangzhou, China), then incubated for 30 min, and finally tested using a FACSCalibur Flow Cytometer (BD Biosciences, San Jose, CA, USA). Cell cycle profiles were analyzed by Modifit software (BD Biosciences).

### Cell apoptosis analysis

Cells were trypsinized (without EDTA) into single cells and resuspended with the binding buffer. Cells were then preserved at room temperature and protected from light for 15 min after staining with the Annexin V-FITC/PI apoptosis kit (Multi Sciences, Hangzhou, China). Cell apoptosis profiles were generated using a FACSCalibur Flow Cytometer (BD Biosciences) with FlowJo 7.6.1 software.

### Cell migration

Cell migration was measured by wound healing analysis and transwell migration assays. In the wound healing analysis, cells were plated in the 6-well plates after scratching the plate with a pipette tip and photomicrographs of wounds were taken by a microscope (Olympus, Japan) at 0 h. Then, cells were then cultured with serum-free medium for 24 h and photomicrographs were taken of the same view. ImageJ software (National Institutes of Health, Bethesda, Maryland, USA) was used to measure the wound area at 0 and 24 h. To generate more convincing results, a transwell assay was performed. In the upper chamber, cells were incubated with serum-free medium. Medium containing 10% FBS was added into the lower chamber. After 24 hours, migrated cells were on the outer side of the chamber membrane and it was then fixed with 4% paraformaldehyde and stained with 0.1% crystal violet (Sigma, Germany).

### Western blot assay

Cells were lysed in a RIPA lysis buffer (Beyotime, Haimen, China) to extract total protein. Protein was separated on 12% SDS-polyacrylamide gels and transferred onto polyvinylidene fluoride (PVDF) membranes (Millipore, Billerica, MA, USA). Skimmed milk was used for blocking PVDF membranes. Immunoblotting of the membranes was conducted by incubation with primary antibodies: P27, CyclinD1 (CST, Danvers, MA, USA) and GAPDH (Affinity Biosciences, Cincinnati, OH, USA). Immunoblotting was carried out on a shaking table at 4 °C overnight. After incubation with a second antibody (CST) for one hour at room temperature and washing with TBS-T, a Western Bright ECL HRP substrate (Advansta, Menlo Park, CA, USA) was added to the membrane and it was visualized by an automated chemiluminescence image analysis system (Tanon 5200, Shanghai, China). Densities of protein bands were measured using ImageJ software (NIH, Bethesda, USA).

### Dual-luciferase reporter assay

To examine whether AC026166.2-001 has ceRNA activities in LSCC, we predicted lncRNA target sites using the online microRNA-target program (http://www.mirdb.org, http://www.mircode.org). According to Watson-Crick complementary characteristics, AC026166.2-001 was predicted to interact with many microRNAs (miRNAs). The GP-miRGLO Dual-Luciferase vector (Promega, Madison, WI, USA) AC026166.2-001 wild and mutant-types (WTs and MUTs) were designed and constructed by GenePharma (Shanghai, China). GP-miRGLO vector constructs (AC026166.2-001 WT or AC026166.2-001 MUT) were cotransfected with miRNA mimics or negative controls in 293 T cells using Lipofectamine 2000 (Thermo Fisher Scientific, Waltham, MA, USA). After 24 hours, cells were washed and treated with the Dual-Luciferase^®^ Reporter Assay System (Promega, Madison, WI, USA) according to the manufacturer’s instructions. Luciferase activity was measured by a multifunctional microplate reader (SpectraMax M5, Molecular Devices, California, USA).

### Xenograft mouse model

All animal studies were conducted using animal protocols approved by the Institutional Animal Care and Use Committee of the Zhejiang Academy of Traditional Chinese Medicine in accordance with institutional guidelines. Male BALB/c nude mice at an age of 4 weeks were purchased from the Sippr-BK Laboratory Animal Co. Ltd. (Shanghai, China) and raised in the laboratory animal research center at Zhejiang Academy of Traditional Chinese Medicine. BALB/c nude mice were inoculated with a total of 200 μl of AMC-HN-8 cell suspension (at a concentration of 5 × 10^7^ cells/ml). After 10 days, nude mice were randomly divided into 4 groups (8 mice per group). A high-dose group of mice was injected with 100 μl of LV5-AC026166.2-001 (5 × 10^8^ TU/ml) and a low-dose group was injected with 100 μl of LV5-AC026166.2-001 (5 × 10^7^ TU/ml). The NC group was injected with 100 μl of LV5-NC (5 × 10^7^ TU/ml). Mice were injected for 3 consecutive days. Blank controls represent non-injected control animals. Weight and tumor volume were measured every two days. Mice were sacrificed 28 days after the last injection and solid tumors were prepared for histopathologic examination.

### Hematoxylin-eosin (HE) staining

Tumor tissues were fixed in 10% formalin, embedded in paraffin and then cut into 8-µm-thick sections that were baked at 45 °C for 5 h. Sections were then stained with HE (artificial hematoxylin and eosin) with the following steps: 30 min of xylene dewaxing, treated with ethanol at different concentrations (100%, 90%, 70%), hydrated in distilled water, stained with hematoxylin (15 min), differentiated in hydrochloric acid ethanol and ammonia water, dehydrated with ethanol at 70% and 90% concentrations (10 min), stained with eosin ethanol (3 min), dehydrated with ethanol and cleared with xylene, and tumor tissue sections were then observed under a microscope.

### Transmission electron microscopy

Transmission electron microscopy (Hitachi H-7650, Tokyo, Japan) was used for investigating apoptotic morphological changes of cells. Tumor tissues were fixed with 2.5% glutaraldehyde for 1 h, then rinsed in phosphate buffer for 1 h. Next, tumor tissues were fixed 30 min to 1 h with 1% osmium acid and dehydrated in a graded ethanol and acetone series. Finally, tissue was embedded in epoxy resin and stained with uranyl acetate and lead citrate.

### TUNEL staining

TUNEL staining was performed using the *in situ* Colorimetric TUNEL Apoptosis Assay Kit (Beyotime Institute of Biotechnology, Beijing, China) according to the manufacturer’s instructions. Briefly, the paraffin embedded tissue sections were washed two times with xylene (10 min), dehydrated with ethanol from high to low concentrations, then slices were pretreated with or without DNase proteinase K (20 mg/mL; Millipore, Boston, MA, USA) at 37 °C for 20 minutes. Sections were then washed 3 times with PBS and incubated in 3% hydrogen peroxide solution (3% H_2_O_2_ in PBS) at room temperature for 20 minutes. Then, 50 L of Streptavidin-HRP working fluid was added to the sample and it was incubated at room temperature for 30 minutes and washed 3 times with PBS. A total of 300 μl of diaminobenzidine (DAB) solution was added, incubating at room temperature for 10 minutes and positive cells were analyzed under light microscopy. The percentage of apoptotic cells was scored as an average of the ratio of TUNEL-positive (TUNEL+) cells to the total number of cells present in each field.

### Statistical analysis

Data were characterized as the mean ± SD (standard deviation). SPSS 18.0 (SPSS, Inc., Chicago, IL, USA) was used for statistical analysis, differences between two groups were analyzed using Student’s *t*-test and were defined as statistically significant for *P* values (obtained from two-sided analysis) < 0.05.

## Electronic supplementary material

Supplementary information
