# Supplementary material for: Long non-coding RNA AC026166.2-001 inhibits cell proliferation and migration in laryngeal squamous cell carcinoma by regulating the miR-24-3p/p27 axis
Source: Sci Rep. 2018 Feb 20;8:3375. doi: 10.1038/s41598-018-21659-5 (PMC5820272; doi:10.1038/s41598-018-21659-5)
Supplement: Supplementary file 1 — Supplementary information [file 41598_2018_21659_MOESM1_ESM.pdf]

Long non-coding RNA AC026166.2-001 inhibits cell proliferation and migration in laryngeal squamous cell carcinoma by regulating the miR-24-3p/p27 axis

Zhisen Shen[1,2*], Wenjuan Hao[1,2], Chongchang Zhou[1,2], Hongxia Deng[1], Dong Ye[2], Qun Li[2], Lexi Lin[2], Bing Cao[2], Junming Guo[1*]

Supplementary data

Supplementary figure

Figure S1

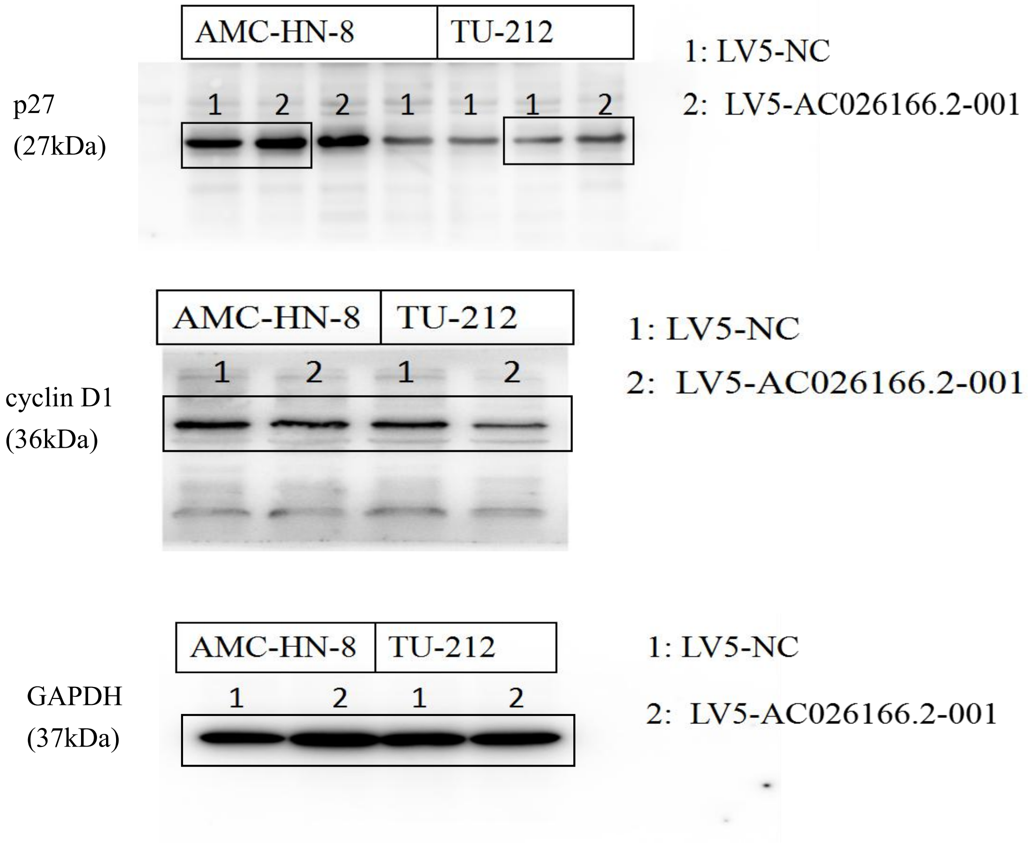

Figure S1. Protein levels of p27, cyclin D1 in AMC-HN-8 and TU-212 cells.
